# Factors related to out-of-hours help-seeking for acute health problems: a survey study using case scenarios

**DOI:** 10.1186/s12889-018-6332-6

**Published:** 2019-01-08

**Authors:** Ellen Keizer, Morten Bondo Christensen, Anders Helles Carlsen, Marleen Smits, Michel Wensing, Oliver Senn, Linda Huibers

**Affiliations:** 1 Scientific Center for Quality of Healthcare (IQ healthcare), Radboud Institute for Health Sciences, Radboud University Medical Center, P.O. Box 9101, 6500 HB Nijmegen, the Netherlands; 2 Institute of Primary Care, University of Zurich and University Hospital of Zurich, Pestalozzistrasse 24, 8091 Zurich, Switzerland; 3 Research Unit for General Practice, Aarhus, Bartholins Alle 2, DK-8000 Aarhus, Denmark

**Keywords:** After-hours care, Emergency medical services, Primary health care, Help-seeking behavior

## Abstract

**Background:**

The acute out-of-hours healthcare services are challenged by increasing demand in many countries. We aimed to examine factors influencing the intended help-seeking in out-of-hours care for acute health problems during evenings, nights, and weekends.

**Methods:**

We conducted a survey study based on data from parents of children (aged 0–4 years) and adults (aged 30–39 and 50–59 years) in Denmark, the Netherlands and Switzerland. Intended help-seeking behaviour was measured by six hypothetical case scenarios. We used Andersen’s Behavioural Model to categorise potentially influential factors and applied multiple binomial regression to assess the influence of selected factors.

**Results:**

A total of 1015 parents and 2942 adults participated. We identified several significant influential factors. Parents holding a low education (OR 1.56), having migrant background (western: OR 1.23; non-western: OR 1.93), having one child (OR 1.24), perceiving few barriers to using out-of-hours primary care (OR 1.59), perceiving difficulties with organising childcare (OR 1.13), and having a history of frequent contacts with out-of-hours care (OR 1.55) were more inclined to contact out-of-hours care, whereas female (OR 0.85) and non-anxious parents (OR 0.77) were less inclined. Adults who were older (OR 1.01), holding a medical education (OR 1.13), having non-western background (OR 1.28), being unemployed (OR 1.17), perceiving few barriers to using out-of-hours primary care (OR 1.37), and having a history of frequent contacts with a GP (few: OR 1.15; more: OR 1.22) and/or with out-of-hours care (one: OR 1.20; more: OR 1.49) were more inclined to contact out-of-hours care, whereas adults with no or little social support (OR 0.84) and adults with high health literacy level on health information (OR 0.91) were less inclined. Dutch parents were less inclined than Danish parents to contact out-of-hours care (OR 0.62), whereas Swiss adults were more inclined than Danish adults to contact out-of-hours care (OR 1.16).

**Conclusion:**

We identified several factors related to intended help-seeking in out-of-hours care. These results could be used to develop targeted interventions, but more research is needed to examine the underlying explanations for the identified differences.

**Electronic supplementary material:**

The online version of this article (10.1186/s12889-018-6332-6) contains supplementary material, which is available to authorized users.

## Background

An increasing number of individuals seek help at the acute out-of-hours services; this trend is seen in many countries [1, 2]. Individuals differ in their help-seeking behaviour when experiencing a health problem. Some have a high threshold for requesting medical care, whereas others contact for harmless conditions. Worry is one of the main reasons for patients to contact out-of-hours care for non-urgent problems and is especially seen among parents of young children [3, 4]. Prompt help-seeking could lead to early detection of disease and prevent aggravation of the health problem. Conversely, healthcare seeking is frequently assessed as potentially inappropriate from a medical viewpoint in acute healthcare settings. Potentially inappropriate healthcare use has also been suggested to be one of the reasons for overcrowding in the emergency departments (EDs) at hospitals and the high demand for services in out-of-hours primary care [2, 5]. At the ED and ambulance services, a substantial part of the patients could instead have been treated by a general practitioner (GP) [6, 7]. Additionally, many of the patients requesting out-of-hours primary care could have waited and contacted their own GP in the regular consultation hours, or the situation could have been handled by self-care [3, 8].

Many factors influence help-seeking behaviour, including the characteristics of the individual patient and the organisation of the healthcare system [9]. Andersen’s Behavioural Model of Health Services Use introduces three key elements that affect healthcare use: predisposing, enabling, and need [10]. Predisposing factors are conditions that are present before an illness occurs, for example demographic factors like age and gender. Enabling factors facilitate or obstruct the healthcare use, for example travel time. Need factors refer to the immediate reasons that lead to the request of healthcare services, for example the individual’s current health status. This behavioural model has been used in many studies and in various settings, including the emergency care setting [11, 12]. Yet, to our knowledge, this framework has not been used for studying help-seeking behaviour in the out-of-hours care setting.

Several differences in help-seeking behaviour also exist between countries, including variations in public expectations and cultural background [9, 13]. Mapping the impact of individual differences and variations between countries in help-seeking behaviour could provide valuable insight into the increasing demand for out-of-hours healthcare. Identification of factors related to frequent use of out-of-hours care could also inform future interventions aiming for more medically appropriate use of available resources in out-of-hours healthcare.

The objective of this study was to examine factors influencing intended help-seeking in out-of-hours care for acute health problems outside regular hours, i.e. during evenings, nights, and weekends.

## Methods

### Design and population

We conducted a survey study from December 2015 to January 2016 among individuals in Denmark, the Netherlands, and Switzerland using hypothetical case scenarios. The study formed part of a project undertaken by the European Research Network for Out-Of-Hours Primary Health Care (EurOOHnet) [14]. In addition to the present paper, two other papers have been written: one on clinical out-of-hours help-seeking behaviour in Denmark, the Netherlands and Switzerland [13] and one on the impact of alternative healthcare plans on intended out-of-hours help-seeking in Switzerland [15].

We included individuals of three age groups: parents of children aged 0–4 years, adults aged 30–39 years, and adults aged 50–59 years. We chose these age groups because a previous study found differences in the use of out-of-hours care between Danish and Dutch young children and young adults [16]. We added the age group 50–59 years to include a broader range of age groups. We aimed to get 600 respondents per age group per country, to gain enough power for one of the other studies [13]. Due to different data collection methods and expected variations in response rates, we selected a different number of individuals for each country. For Denmark, we used the Danish Civil Registration System, which holds information on all Danish individuals, to select 1200 individuals per age group. Individuals living in institutions and individuals with unknown address were excluded. For the Netherlands and Switzerland, a nationally representative consumer panel was used for each country. For the Netherlands, we used the consumer panel of TNS NIPO, a professional organisation for market research, to select 950 individuals per age group. This consumer panel consists of a representative group (over 200,000 members) of citizens (www.tnsglobal.com, 2017). For Switzerland, 6093 representative German-speaking members of two consumer panels (Respondi and Bilendi) were used to select 600 respondents for the two adult age groups.

### Setting

The organisation of the healthcare system in Denmark and the Netherlands is quite similar. Almost all Danish and Dutch citizens are listed with a GP, who acts as a gatekeeper to secondary care. Outside office hours, a GP cooperative can be contacted by telephone. Self-referral to the ED is possible, but is generally discouraged as it is mostly preferable to first contact primary care. Primary care is free of charge in both daytime and outside office hours. An ED visit is free of charge in Denmark, whereas residents in the Netherlands must pay an annual (tax-deductible) fee of at least EUR 375 (2015 figures).

In Switzerland, patients can freely access the ED and specialist care. However, patients may choose another healthcare insurance plan, which reduces the costs but also obligates them to first contact a gatekeeper (for example a GP) for healthcare. The organisation of out-of-hours care varies between the regions. GP and emergency care is covered by the mandatory health insurance plan, except for an annual (tax-deductible) fee of at least CHF 300 (approx. EUR 275) and 10% co-payment [15].

### Questionnaires

We developed two questionnaires for the study: one for parents of young children and one for adults. Both questionnaires consisted of predefined cases describing situations involving specific symptoms and signs of disease. After the cases, respondents were asked to answer questions concerning factors related to help-seeking behaviour based on Andersen’s Behavioural Model. The cases for parents and adults differed, but all cases described situations that could prompt a need for acute healthcare. All cases involved frequently occurring health problems at different levels of urgency. An English version of the questionnaires are presented in Additional files 1 and 2**.**

### Development of case descriptions

To ensure that the presented cases constituted sufficient content validity, the development process consisted of several steps. We selected previously used cases from other studies [17–19] and added new cases at different levels of urgency inspired by common reasons for encounter in the three included countries. Each case described a situation, including a specific weekday and time. For cases involving children, we stated a specific age of the child as even small age differences in this group can change the intended help-seeking behaviour in the parents (even for the same illness). For cases involving adults, we did not state a specific age, but we gave an age range (30–39 years or 50–59 years) to ensure that the respondents were able to see themselves in the described situation. The cases underwent several feedback cycles (both face-to-face and by email) with researchers, GPs, and laypersons. Finally, we ended up with 20 cases concerning children and 32 cases concerning adults.

To get an overview of the urgency levels of the cases and to check the representativeness and clarity of formulations, an expert panel of 29 GPs reviewed the cases. These GPs had to meet the following inclusion criteria: ≥ 2 years GP, ≥ 6 out-of-hours shifts per year, coming from different regions within the countries, and fair knowledge of English. Cases classified as ‘unclear’ according to the expert panel were excluded. In a research meeting, we selected 11 cases concerning children and 13 concerning adults with different levels of urgency. The included cases were translated from English into Danish using a backward-forward translation procedure and a consensus meeting [20]. The cases were sent to 150 Danish individuals per age group and tested for variations in help-seeking behaviour. We performed a Rasch analysis and selected cases across the whole range, and cases without response variation were excluded. This resulted in a final selection of cases representing varying responses; six cases for children and six cases for adults.

### Outcome measure: Intended help-seeking behaviour

The six cases were used to measure our outcome measure “intended help-seeking behaviour outside office hours”. For each case, we dichotomised the individual responses concerning intended behaviour into “Yes/1” and “No/0” categories: ‘*Contacting out-of-hours care*’ (‘Contact out-of-hours primary care’, ‘Contact the ED’, ‘Contact 112/144 ambulance care’) and ‘*Not contacting out-of-hours care’* (‘Wait-and-see’, ‘Self-care’, ‘Ask my partner, a relative, or others for advice’, ‘Check a guidebook, the internet, or an app’, ‘Contact my own GP on the next working day’). Intended help-seeking behaviour was estimated by combining the dichotomised scores of the six cases for each respondent.

### Theoretical framework and development of model

**Fig. 1:**
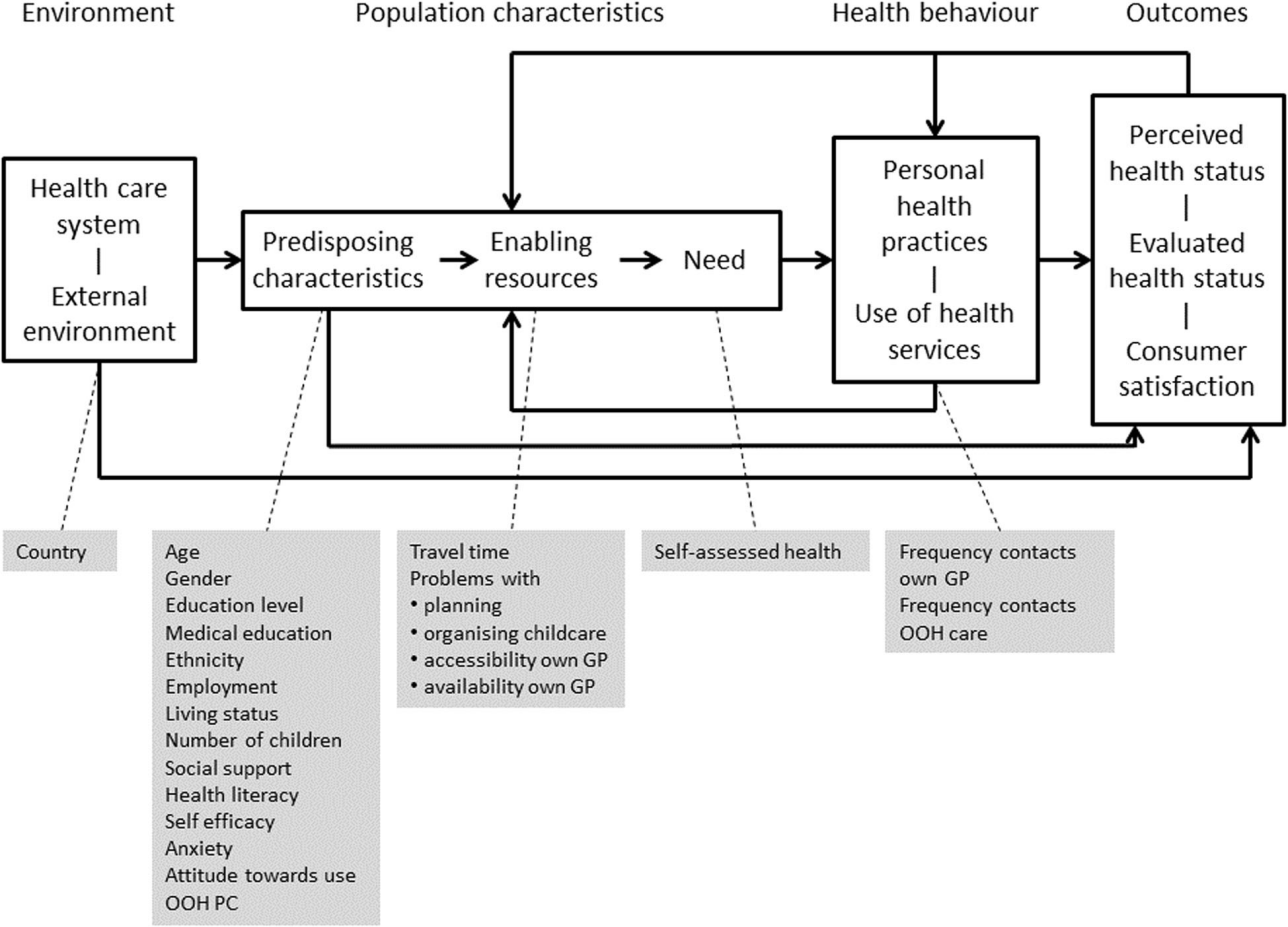
Model of help-seeking behaviour with included variables based on Andersen’s Behavioural Model [10]

The study was guided by Andersen’s Behavioural Model of Health Services Use [10], which defines population characteristics (predisposing characteristics, enabling resources, and need), health behaviour, and outcomes that may affect the use of health services **(**Fig. 1**)**.

The following predisposing characteristics were included: age, gender, education level, medical education, ethnicity, work status, living status, number of children (for parents of children aged 0–4 years), social support, health literacy (navigating the system and finding information), self-efficacy, anxiety, and attitude towards use of out-of-hours primary care. The following enabling factors were included: travel time, problems with planning, organising childcare (for children), and accessibility and availability of own GP. We included one need factor (self-assessed health of adult and child), two behavioural factors (frequency of contacts with own GP and frequency of contacts with out-of-hours care), and one environment factor (country). For some of the determinants, the following validated questionnaires were used: Generalized Anxiety Disorder scale (GAD-2) [21], General Self-Efficacy Scale (GSE-10) [22], two scales from the Health Literacy Questionnaire (HLQ) [23], and the self-assessed health item from the 36-item Short-Form Health Survey (SF-36) [24]. Questions from previous studies were used; sometimes in adjusted form (i.e. on education, employment [25], and social support [26]).

We also added newly developed questions (medical education, living status, attitude towards use of out-of-hours primary care, and perceived problems). For the Netherlands and Switzerland, standard questions for the internet panels were used (age, gender, education, and employment). The data preparation of these factors is described in Additional file 3.

### Interviews and pilot study

The readability and feasibility of the original Danish version of the questionnaire were tested in two steps. First, cognitive interviews with eight patients from one GP practice were conducted to see if they understood the questions. Second, we performed a pilot study by sending the questionnaire to 50 Danish individuals per age group, including one reminder. The pilot study resulted in minor layout adjustments and showed good feasibility with a response rate of 38% for children, 28% for adults aged 30–39 years, and 50% for adults aged 50–59 years.

### Data collection

The Dutch and Swiss questionnaires were each translated from the Danish source text by using the backward-forward procedure and a consensus meeting [20]. The Danish individuals received a paper questionnaire in January 2016 with the option to complete the questionnaire online, and a reminder was sent three weeks later. The Dutch consumer panel members received an email with a link to the questionnaire in December 2015, and a reminder was sent for age groups 0–4 and 30–39 years (the aimed response rate was met for age group 50–59 years). The data collection stopped after one week as the minimum of 600 respondents had been reached for all groups. The Swiss consumer panel members were invited by an email link in December 2015; all 6093 individuals in the age groups 30–39 and 50–59 years were contacted. The data collection stopped after five days as the minimum of 600 respondents per age group had been reached. The datasets received from the consumer panel organisations included only anonymous data. The Danish respondents participated in a draw for three sets of two cinema tickets, whereas the Dutch and Swiss consumer panel members each received a small financial compensation as a standard procedure.

### Statistical analysis

We checked the representativeness of our data. For Denmark, we compared respondents with non-respondents for age, gender, region, education level, ethnicity, living status, and employment as our sample was selected randomly from the entire population. For all countries, we compared respondents with the general population (age, gender, region, education level, ethnicity, living status, and employment) using 95% confidence intervals (CI).

All analyses were done separately for children and adults (adults consisted of two age groups). Descriptive analyses were used to show the distribution of factors affecting help-seeking behaviour. Two multiple binomial regression analyses were performed to assess the influence of all factors on the inclination to contact out-of-hours care (one for parents and one for adults). Odds ratios (ORs) were calculated and presented in forest plots with 95% confidence intervals. For all analyses, we combined data from all participating countries (Denmark and the Netherlands for cases based on children; all three countries for cases based on adults). We performed the binomial regression analyses separately for each country to check the robustness of our data (data not presented). All analyses were conducted using Stata 14.2 (StataCorp LP, College Station, TX, USA).

## Results

### Population

In Denmark, we obtained responses from 572 parents of children (47.7%), 429 adults aged 30–39 years (35.8%), and 652 adults aged 50–59 years (54.3%). The overall response rate was 44.2% for adults. In the Netherlands, we ended the data collection after one week as the required number of completed questionnaires had been reached: 621 responses from parents of children, 592 from adults aged 30–39 years, and 633 from adults aged 50–59 years. In Switzerland, the data collection also ended when the aim of approximately 600 respondents per age group had been reached: 589 responses from adults aged 30–39 years and 595 from adults aged 50–59 years.

Table 1 shows the characteristics of the study population. When checking for representativeness, we found that respondents were slightly higher educated (except for the Swiss population aged 50–59 years), more often native, more often female, and less often living alone than the general population in the three countries (data not shown).

**Table 1:** Description of study population (%)

	Factors	Categories	Parents (N_max_ = 1193)^a^	Adults (N_max_ = 3490)^b^
Predisposing	Age, mean (SD)		34.9 (5.1)	45.4 (10.2)
	Gender	Male	26.6	46.5
		Female	73.4	53.5
	Education level	Low	5.7	11.9
		Middle	31.7	51.9
		High	62.5	36.1
	Medical education	None	84.7	90.1
		Some/nurse/doctor	15.3	9.9
	Ethnicity	Native	83.5	79.1
		Western migrant	8.7	15.8
		Non-western migrant	7.7	5.1
	Employment	Unemployed	23.5	20.1
		Employed	76.5	79.9
	Living status	Living alone	4.3	17.0
		Living with another adult	95.7	83.0
	Number of children	One	25.6	n.a.
		More than one	74.4	
	Social support	Lacking social support	15.4	25.7
		Receiving social support	84.6	74.3
	Health literacy – navigating the system	Low ability	4.0	5.0
		Middle ability	24.1	23.9
		High ability	58.3	55.3
		Highest ability	13.6	15.7
	Health literacy – finding information	Low ability	8.3	9.7
		High ability	71.1	71.1
		Highest ability	20.6	19.2
	Self-efficacy	Low	53.5	49.3
		High	46.5	50.7
	Anxiety	No anxiety	92.7	87.9
		Anxiety	7.3	12.1
	Attitude towards use of out-of-hours primary care	Low barrier	37.8	40.1
		High barrier	62.2	59.9
Enabling	Travel time	< 15 min	49.4	47.1
		15–30 min	43.0	43.2
		> 30 min	7.7	9.7
	Problems – own work	No/few problems	75.1	83.6
		Some/many problems	24.9	16.4
	Problems - organising childcare	Easy	44.4	n.a.
		Difficult	55.6	
	Problems - accessibility of own GP	No/few problems	66.3	79.1
		Some/many problems	33.7	20.9
	Problems - availability of own GP	No/few problems	77.4	84.8
		Some/many problems	22.6	15.2
Need	Self-assessed health (child/adult)	Poor	2.6	13.7
		Good	97.4	86.3
Behaviour	Frequency of contacts to own GP	None/one contact	11.9	39.2
		Few contacts	47.9	43.3
		More contacts	40.2	17.5
	Frequency of contacts to out-of-hours care	None	27.3	66.8
		One contact	24.5	17.3
		More contacts	48.2	15.8

*N.a* not applicable

^a^Percentage of missing values ranged from 0% (age, gender) to 5.3% (frequency of out-of-hours care)

^b^Percentage of missing values ranged from 0% (age, gender) to 5.1% (travel time)

### Factors influencing intended help-seeking behaviour

**Fig. 2:**
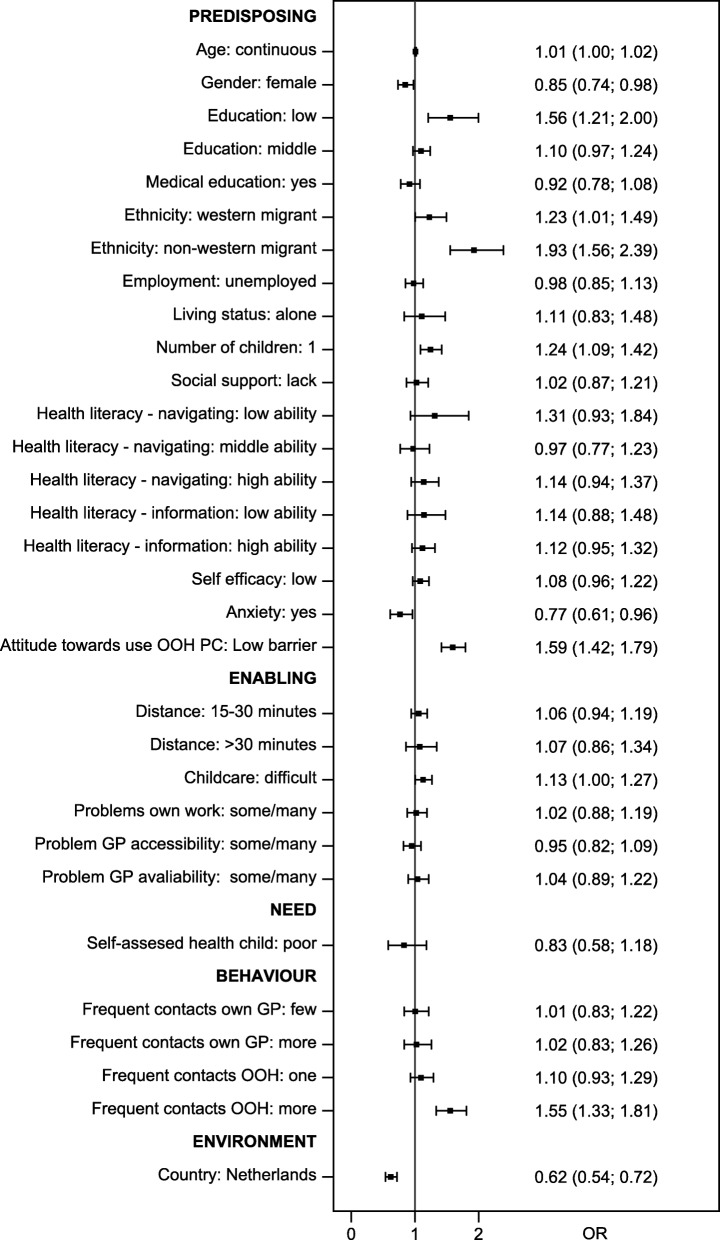
Help-seeking behaviour of parents (OR and 95% confidence level)

Figure 2 presents factors related to intended help-seeking behaviour concerning children (*N* = 1015). We found that women were less inclined than men to contact out-of-hours care for their child (OR 0.85, 95% CI 0.74–0.98), and low educated parents had higher probability of seeking help than high educated parents (OR 1.56, 95% CI: 1.21–2.00). Furthermore, parents with a migrant background were more inclined to seek help for their child than parents with native background (western: OR 1.23, 95% CI 1.01–1.49; non-western: OR 1.93, 95% CI 1.56–2.39). Parents with one child also tended to contact out-of-hours care more frequently than parents with more than one child (OR 1.24, 95% CI 1.09–1.42). Parents with anxiety were less inclined to contact out-of-hours care than parents without anxiety (OR 0.0.77, 95% CI 0.61–0.96). Parents perceiving few barriers to using out-of-hours primary care were more inclined to contact out-of-hours care for their children than parents perceiving barriers to use out-of-hours primary care (OR 1.59, 95% CI 1.42–1.79). Parents who perceived difficulties in organising childcare were more inclined to contact out-of-hours care than parents who did not perceive such difficulties (OR 1.13, 95% CI 1.00–1.27). Additionally, in comparison with parents who had not used out-of-hours care during the last year, parents who had frequently used out-of-hours care were also more inclined to contact out-of-hours care (OR 1.55, 95% CI 1.33–1.81). Finally, we found a difference between the Danish and the Dutch parents; Dutch parents generally chose to contact out-of-hours care less often than Danish parents did (OR 0.62, 95% CI 0.54–0.72).

**Fig. 3:**
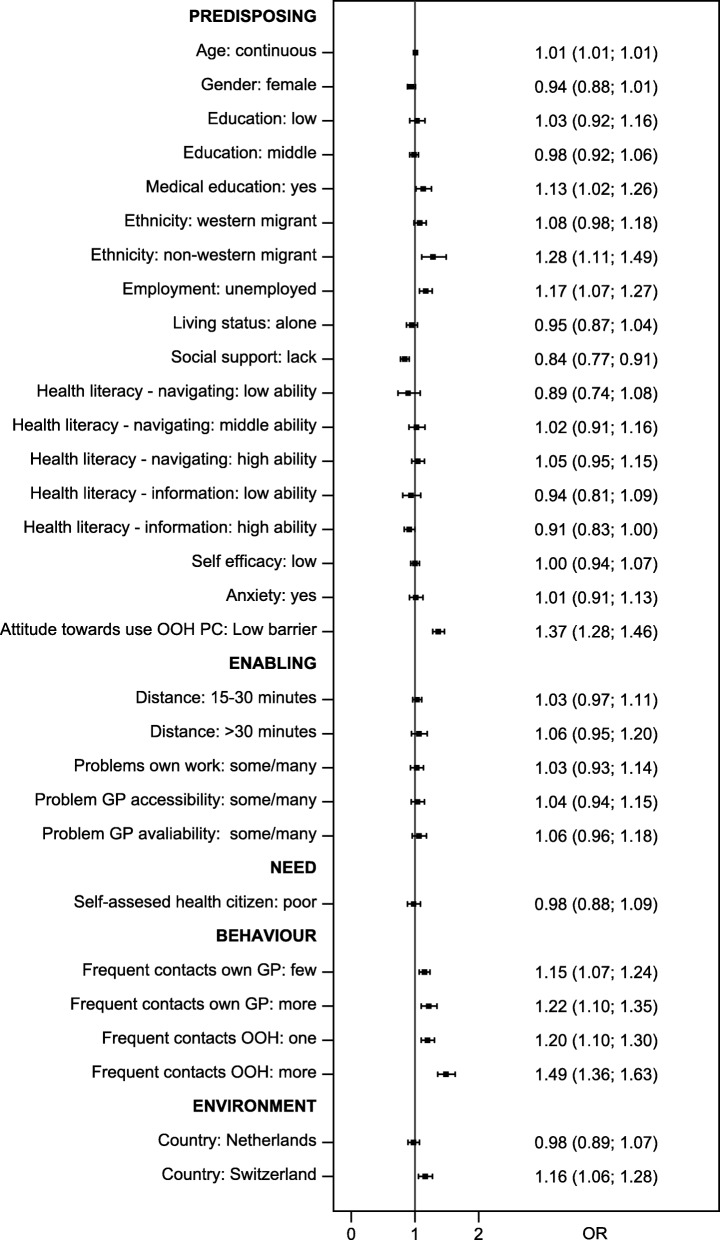
Help-seeking behaviour of adults (OR and 95% conficence level)

Figure 3 shows the results for factors related to intended help-seeking behaviour concerning adults (*N* = 2942). We found that the probability for contacting out-of-hours care increased with age (OR 1.01, 95% CI 1.01–1.01). Individuals with a medical background would more often contact out-of-hours care than individuals with no medical background (OR 1.13, 95% CI 1.02–1.26). Non-western migrants were more inclined to contact out-of-hours care than native individuals (OR 1.28, 95% CI 1.11–1.49), and unemployed individuals had higher probability of seeking help than employed individuals (OR 1.17, 95% CI 1.07–1.27). Individuals with no or low social support were less likely to contact out-of-hours care than individuals with high social support (OR 0.84, 95% CI 0.77–0.91); this trend was even seen for individuals with high health literacy level on health information (OR 0.91, 95% CI 0.83–1.00). We also found that individuals who perceived few barriers to using out-of-hours primary care would more often contact out-of-hours care than individuals who perceived barriers (OR 1.37, 95% CI 1.28–1.46). Individuals who had few or more contacts with their GP were more inclined to contact out-of-hours care than individuals who had no contacts with their GP (few: OR 1.15, 95% CI 1.07–1.24; more: OR 1.22, 95% CI 1.10–1.35). Additionally, individuals who had frequently contacted out-of-hours care previously would more often contact out-of-hours care than those with a history of infrequent contact (one: OR 1.20, 95% CI 1.10–1.300; more: OR 1.49, 95% CI 1.36–1.63). Finally, we found that the Swiss population was generally more inclined to contact out-of-hours care than the Danish population (OR 1.16, 95% CI 1.06–1.28).

Stratified analyses per country showed the same significant associations for most of the help-seeking factors (data not shown). Some associations were no longer present after stratification (most likely due to lack of power), but we still found trends and associations in the same direction as for the overall data.

## Discussion

### Main findings

For parents, the following predisposing factors were related to higher inclination to contact out-of-hours care for their children: male, low education, migrant, having one child, being non-anxious, and perceiving few barriers to using out-of-hours care. For adults, individuals characterized by older age, medical education, being a non-western migrant, unemployment, having social support, and perceiving few barriers to using out-of-hours care were more inclined to contact out-of-hours care. The enabling factor “problems with organising childcare in case of illness” and the behavioural factors “previous contact with GP care” (for adults) and “previous contact with out-of-hours care” (for adults and parents) increased the inclination to seek help. The environment, which was expressed by the factor “country”, also seemed to influence the help-seeking behaviour: Danish parents were more inclined to contact out-of-hours care than Dutch parents, and Swiss adults were more inclined to contact out-of-hours care than Danish adults.

### Interpretation of results and comparison with literature

We found that older individuals, low educated individuals, and non-western migrants were more inclined to contact out-of-hours care, which has also been reported in other studies [9, 27]. However, these previous studies showed that women were more inclined to contact healthcare, whereas we found that women were less inclined to contact out-of-hours care for their child. Men and women might react differently when it comes to their child than when a health issue concerns themselves. This could be related to the traditional caretaker role of women, which could make men less certain about childhood diseases and thus more likely to contact medical experts.

In line with our findings, a previous study found that parents with one child were more inclined to seek help than parents with more children [28]. These parents may be more easily worried because they have limited experience with common childhood diseases, such as fever, and thus may seek advice sooner. Apart from “organising childcare in case of illness”, we did not find any effects of need and enabling factors, such as health status, distance to healthcare services, and access to daytime general practice, although other studies have reported some influence [9, 29–31]. One explanation could be the extensive model that we used; the effect of some of the included factors, such as access to daytime general practice, could possibly be influenced by other factors, such as ethnicity and education. We found that adults with social support were more inclined to contact out-of-hours care. An explanation could be that people from the lay referral network may encourage contact with a doctor in an acute situation or in case of doubt [32].

Our analyses showed that non-anxious parents were more inclined to contact out-of-hours care. We expected to find the opposite because we hypothesized that anxious parents would get worried more quickly and thus would be more likely to contact out-of-hours care frequently. It is difficult to explain this result, but high anxiety could make it more difficult to contact healthcare, or these parents may feel uncomfortable with contacting unknown doctors.

Although we have used a comprehensive model allowing for many factors, other factors could also have an important role. For example, other studies have shown some influence of chronic and mental disease and some impact on the quality of the communication with the GP [9, 31, 33]. Other unknown factors could also be relevant, and some factors may affect only subgroups. The most obvious factors that could influence help-seeking behaviour are probably need factors, such as the type and characteristics of the health problem.

A previous study found that Danes more often contacted out-of-hours primary care than the Dutch [16], which is in line with our findings for children. Another study on the propensity to seek healthcare in 34 different countries found that Denmark scores highest on contacts for minor complaints [9]. The influence of the factor “country” is difficult to interpret; both differences related to culture and to the healthcare system could be relevant. Additionally, other factors that were not included in our model could also play a role. One of the explanations for the difference between Danish and Dutch parents could be the direct access to a GP who answers the telephone in Danish out-of-hours primary care (whereas a nurse performs the triage in the Dutch out-of-hours setting).

### Strengths and limitations

A strength of this study is that we studied the intended help-seeking behaviour of a broad range of individuals, including those who never consulted a GP or an ED. We included a number of relevant potentially influential factors, which were adjusted for each other. We also presented a fairly complete overview of relevant factors that could influence intended help-seeking behaviour, and the theoretical framework was based on Andersen’s acknowledged behavioural model. The case descriptions were systematically developed and pilot tested.

One of the limitations of our study is that we used paper-based case scenarios to measure help-seeking behaviour. Asking about behaviour in hypothetical situations may not represent actual behaviour and could include social desirability bias. We cannot rule out the possibility that individuals make other decisions in real life. Nevertheless, help-seeking behaviour is mainly determined by behavioural intentions [34]. Help-seeking behaviour was measured by combining the decisions in six cases, and the selection of cases could have influenced the results found. Yet, we believe that this would have mainly affected our effect size rather than the direction of findings. Factors related to help-seeking could also differ according to case or between medically appropriate and inappropriate use. A previous study has shown that the association between gender and help-seeking behaviour depends on the symptom studied [35]. Furthermore, although our response rates were acceptable for this type of study, we cannot rule out selection bias. In addition, different recruitment methods were used to include citizens from the three countries. Consumer panels were used in the Netherlands and Switzerland, which may have introduced some bias since our respondents did not completely represent the general population. However, as our study focused on associations between influential factors and help-seeking, we believe that this has not substantially affected our results. Moreover, the fact that respondents were incentivised with a small amount of money or a chance to win a cinema voucher could have introduced some bias. Still, this approach could also have resulted in a more representative sample since groups that are known to have lower responsiveness may feel encouraged to participate in this study [36]. Finally, the questionnaire was only pilot tested in Denmark, which may have resulted in lower readability for the Dutch and Swiss participants. However, we expect to have addressed the most important readability issues in the general phrasing of questions, which was taken into account when translating the questionnaire into Dutch and German using the recognised forward-backward translation procedure.

### Clinical implications and future research

We found several factors that were related to a higher intended use of out-of-hours care, and some of these could be included in interventions aiming to ensure optimal use of out-of-hours care. For example, perceiving few barriers towards the use of out-of-hours primary care seems to be an important factor for help-seeking and may lead to medically inappropriate use. It may be possible to educate individuals about the purpose of out-of-hours primary care; this could be done during the contact with an out-of-hours primary care service, during the contact with own GP, or through a nationwide patient education campaign. Yet, the effectiveness of patient education is debatable [37]. Furthermore, parents with one child were found to have higher use, and other information sources targeting this group (such as apps or help lines) could be investigated [3, 38].

We studied intended help-seeking, regardless of its appropriateness. Yet, we cannot rule out that factors associated with help-seeking behaviour may differ between citizens with inappropriate and citizens with appropriate help-seeking. Future research could examine influential factors related to potentially inappropriate help-seeking. Furthermore, the factor “country” was found to influence the help-seeking of some individuals, but we could only speculate about the explanations for these differences. Future research could focus on the effectiveness of healthcare systems and the prevailing help-seeking culture. Since intended help-seeking behaviour in out-of-hours care could vary not only because of different influential factors but also because of different types of healthcare providers, a future study exploring factors associated with contacting primary and secondary healthcare services could be relevant.

The question is whether it is possible to change the help-seeking behaviour in the modern consumer societies, where most individuals expect 24/7 access to a wide range of services. Alternative ways of providing out-of-hours healthcare could be considered, such as evening consultations in general practice.

### Conclusion

*Predisposing* factors (like age, gender, ethnicity, education, employment, number of children, anxiety, social support, health literacy, and attitude towards use of out-of-hours care), *enabling* factors (organising childcare) and *behavioural* factors (previous contact with GP and out-of-hours care) are all factors that influence the intended help-seeking in out-of-hours care. The resident country of the contacting individual also seems to influence the intended help-seeking behaviour. Some of the provided information could be used to develop targeted interventions, but more research is needed to examine the underlying explanations for the identified differences.

## Additional files

Additional file 1: Questionnaire for parents. (DOCX 43 kb)

Additional file 2: Questionnaire for adults. (DOCX 43 kb)

Additional file 3: Categorisation of influential factors. (DOCX 24 kb)

## Abbreviations

CI: Confidence Interval
ED: Emergency Department
GP: General Practitioner
NA: Not applicable
