# Supplementary material for: Factors related to out-of-hours help-seeking for acute health problems: a survey study using case scenarios
Source: BMC Public Health. 2019 Jan 8;19:33. doi: 10.1186/s12889-018-6332-6 (PMC6323727; doi:10.1186/s12889-018-6332-6)
Supplement: Supplementary file 1 — Questionnaire for parents. (DOCX 43 kb) [file 12889_2018_6332_MOESM1_ESM.docx]

**Additional file 1. Questionnaire for parents**

*In the English version of the questionnaires, the terms out-of-hours primary care, emergency department, and 112 ambulance care are used in the response categories. The wording was culturally adapted in the translated questionnaires to match the services available for the target group.*

**SITUATION DESCRIPTIONS**

We present six fictive situations. Each of the situations describes an invented case including a health problem affecting your **child’s** health occurring outside the office hours of your own GP. Please answer what action(s) you would most likely take in this situation at this moment.

We would like to know what you would choose to do in the given situation (i.e. which actions you would most likely take). You do not have to consider what would be the “right” thing to answer or what other people think you should do.

In the cases we refer to a specific age. We ask you to pretend that your son/daughter is of the age stated in the case.

**Case 1**

*Time:* Saturday at 3 PM.

*Situation:* Your 4-year-old child has had abdominal pain for two days, and the pain is increasing in severity. He has a fever (39.6°C). He has vomited twice today and has not eaten anything for the entire day. He will not drink much. He has a little bit of diarrhoea. You cannot comfort him by reading a book, and he does not want to play by himself*.*

1. **What would you do, at this moment?** *(Please give one or more answers)*

- Wait and see (no contact with a doctor or similar)
- Self-care (for example a pain killer)
- Ask your partner, a relative, or others for advice
- Check a medical reference book, the internet or an app (for example “Patienthåndbogen”/”Moet ik naar de dokter?”)
- Contact own general practitioner the next working day
- Contact the out-of-hours primary care outside opening hours own GP
- Contact the emergency department
- Call 112 ambulance care
- Do something else *Please describe:* ___________________________________________________

**Case 2**

*Time:* Sunday evening at 4 PM.

*Situation:* Your 3-year-old child has a cold and has had red eyes with discharge since two days. He is also sniffing. The eye discharge is yellow, and the eye lids stick together slightly. He is watching television.

1. **What would you do, at this moment?** *(Please give one or more answers)*

- Wait and see (no contact with a doctor or similar)
- Self-care (for example rinse with boiled water)
- Ask your partner, a relative, or others for advice
- Check a medical reference book, the internet or an app (for example “Patienthåndbogen”/”Moet ik naar de dokter?”)
- Contact your child’s own general practitioner the next working day
- Contact the out-of-hours primary care outside opening hours own GP
- Contact the emergency department
- Call 112 ambulance care
- Do something else *Please describe:* ___________________________________________________

**Case 3**

*Time:* Saturday at 3 PM.

*Situation:* Your 15-month-old child has woken after his nap with a temperature of 39.8°C. He already seemed listless before his nap today. He has not vomited, has no diarrhoea and no skin rash. He wants to sit with you and watch television. He does not want to eat anything, but drinks small amounts of cold water.

1. **What would you do, at this moment?** *(Please give one or more answers)*

- Wait and see (no contact with a doctor or similar)
- Self-care (for example a pain killer)
- Ask your partner, a relative, or others for advice
- Check a medical reference book, the internet or an app (for example “Patienthåndbogen”/”Moet ik naar de dokter?”)
- Contact your child’s own general practitioner the next working day
- Contact the out-of-hours primary care outside opening hours own GP
- Contact the emergency department
- Call 112 ambulance care
- Do something else. *Please describe:* ___________________________________________________

**Case 4**

*Time:* Saturday at 3 PM.

*Situation:* Your 2-year-old child wakes up after his nap with red rash across arms, legs, chest and face. The rash is itching. He is alert, is playing as usual and has no other complaints and no fever.

1. **What would you do, at this moment?** *(Please give one or more answers)*

- Wait and see (no contact with a doctor or similar)
- Self-care
- Ask your partner, a relative, or others for advice
- Check a medical reference book, the internet or an app (for example “Patienthåndbogen”/”Moet ik naar de dokter?”)
- Contact your child’s own general practitioner the next working day
- Contact the out-of-hours primary care outside opening hours own GP
- Contact the emergency department
- Call 112 ambulance care
- Do something else *Please describe:* ___________________________________________________

**Case 5**

*Time:* Thursday at 7 PM.

*Situation:* Your 8-month-old child has a fever. Last week, he had a common cold with a fever. He was also coughing. He seemed to recover, but now the fever has returned (temperature: 39.1°C). He does not drink a lot, and he is still coughing. Your child wants to sit with you all the time, but you cannot comfort him.

1. **What would you do, at this moment?** *(Please give one or more answers)*

- Wait and see (no contact with a doctor or similar)
- Self-care (for example a pain killer)
- Ask your partner, a relative, or others for advice
- Check a medical reference book, the internet or an app (for example “Patienthåndbogen”/”Moet ik naar de dokter?”)
- Contact your child’s own general practitioner the next working day
- Contact the out-of-hours primary care outside opening hours own GP
- Contact the emergency department
- Call 112 ambulance care
- Do something else *Please describe:* ___________________________________________________

**Case 6**

*Time:* Sunday at 5 PM.

*Situation:* For one day, your 2-year-old child has had red skin and fluid-filled blisters, mostly on the chest and belly. He is a bit warm (temperature: 38.1°C), complains of a sore throat and generally does not seem fit. He drinks and eats as usual and is as alert as usual.

1. **What would you do, at this moment?** *(Please give one or more answers)*

- Wait and see (no contact with a doctor or similar)
- Self-care (for example a pain killer)
- Ask your partner, a relative, or others for advice
- Check a medical reference book, the internet or an app (for example “Patienthåndbogen”/”Moet ik naar de dokter?”)
- Contact your child’s own general practitioner the next working day
- Contact the out-of-hours primary care outside opening hours own GP
- Contact the emergency department
- Call 112 ambulance care
- Do something else *Please describe:*

___________________________________________________

**FACTORS AFFECTING DECISION-MAKING**

The next questions relate to general factors that may affect decision-making regarding health problems.

1. **We are interested in how you feel about the following statements. Read each statement carefully. Indicate how you feel about each statement.** *(Please mark one answer per statement)*

|  |  | **Not at all true** | **Hardly true** | **Moderately true** | **Exactly true** |
| --- | --- | --- | --- | --- | --- |
| 1. | I can always manage to solve difficult problems if I try hard enough | O | O | O | O |
| 2. | If someone opposes me, I can find the means and ways to get what I want | O | O | O | O |
| 3. | It is easy for me to stick to my aims and accomplish my goals | O | O | O | O |
| 4. | I am confident that I could deal efficiently with unexpected events | O | O | O | O |
| 5. | Thanks to my resourcefulness, I know how to handle unforeseen situations | O | O | O | O |
| 6. | I can solve most problems if I invest the necessary effort | O | O | O | O |
| 7. | I can remain calm when facing difficulties because I can rely on my coping abilities | O | O | O | O |
| 8. | When I am confronted with a problem, I can usually find several solutions | O | O | O | O |
| 9. | If I am in trouble, I can usually think of a solution | O | O | O | O |
| 10. | I can usually handle whatever comes my way | O | O | O | O |

*We used validated Danish, Dutch, and German versions of the Generalized Self-Efficacy scale [1, 2].*

1. **Over the last two weeks, how often have you been bothered by the following problems?**

|  |  | **Not at all** | **Several days** | **More than half the days** | **Nearly every day** |
| --- | --- | --- | --- | --- | --- |
| 1. | Feeling nervous, anxious or on edge | O | O | O | O |
| 2. | Not being able to stop or control worrying | O | O | O | O |

*We used validate Danish, Dutch, and German versions of the Generalized Anxiety Disorder scale (GAD-2) [3, 4].*

**Do you have somebody to talk to if you have problems or you need support?** *(Please only mark one answer)*

No, never or almost never

Yes, sometimes

Yes, often

Yes, always

We used two scales of the validated Health Literacy Questionnaire (HLQ). As the HLQ is copyrighted to Deakin University, publication of the items or scales is not permitted [5].

1. **How severe would your child’s medical problem have to be before you felt it was appropriate to contact …?** *(Please mark one grade per row)*

|  | **Not severe** | | | |  |  |  |  | **Very severe** | | | **Don’t know** |
| --- | --- | --- | --- | --- | --- | --- | --- | --- | --- | --- | --- | --- |
| … your own GP | **0** | **1** | **2** | **3** | **4** | **5** | **6** | **7** | **8** | **9** | **10** | O |
| … OOH primary care | **0** | **1** | **2** | **3** | **4** | **5** | **6** | **7** | **8** | **9** | **10** | O |
| … 112 | **0** | **1** | **2** | **3** | **4** | **5** | **6** | **7** | **8** | **9** | **10** | O |

1. **The following statements concern your considerations for contacting OOH-PC. Please answer to which degree you agree with each statement.** *(Please mark one answer per statement)*

|  |  | **Totally agree** | **Agree** | **Not agree and not disagree** | **Disagree** | **Totally disagree** | **Don’t know** |
| --- | --- | --- | --- | --- | --- | --- | --- |
| 1. | The OOH primary care is intended for all medical problems (including non-urgent problems) that occur outside my GP’s normal opening hours | O | O | O | O | O | O |
| 2. | I can contact OOH primary care at any time, because it is financed by taxation (Denmark)/my insurance (the Netherlands) | O | O | O | O | O | O |
| 3. | I feel more personal barriers in relation to contacting OOH primary care than contacting my own GP during daytime | O | O | O | O | O | O |
| 4. | I carefully consider whether I should contact OOH primary care, because I do not want to disturb the health professionals | O | O | O | O | O | O |

**In the past year, how many times have you contacted the following health care providers regarding yourself and/or your children?** *(Please only mark one cross in each row – if you are unsure, please answer what you think is most accurate)*

|  | **Never** | **1** | **2** | **3** | **4** | **5 or more** | **Don’t know/ not relevant** |
| --- | --- | --- | --- | --- | --- | --- | --- |
| Own GP | O | O | O | O | O | O | O |
| OOH primary care | O | O | O | O | O | O | O |
| Emergency department | O | O | O | O | O | O | O |
| 112 | O | O | O | O | O | O | O |

**How satisfied are you in general with the following health care providers?** *(Please only mark one cross in each row)*

|  | **Very satisfied** | **Satisfied** | **Not satisfied, not satisfied** | **Dissatisfied** | **Very dissatisfied** | **Don’t know** | **Not relevant/ no contact** |
| --- | --- | --- | --- | --- | --- | --- | --- |
| Own GP | O | O | O | O | O | O | O |
| OOH primary care | O | O | O | O | O | O | O |
| Emergency department | O | O | O | O | O | O | O |
| 112 | O | O | O | O | O | O | O |

**During the last two years, have you experienced practical problems in contacting your own GP during day time, due to …** *(Please only mark one cross in each row)*

|  | **No problems** | **Yes, few problems** | **Yes, some problems** | **Yes, many problems** | **Don’t know** | **Not relevant** |
| --- | --- | --- | --- | --- | --- | --- |
| … your own working hours or private appointments? | O | O | O | O | O | O |
| … your GPs telephone accessibility? | O | O | O | O | O | O |
| ...the possibility to make a telephone appointment with your GP? | O | O | O | O | O | O |
| … your GPs availability for a clinic appointment? | O | O | O | O | O | O |
| ...the accessibility to your own GP practice by website (i.e. making a appointment, repeat prescription, asking questions)? | O | O | O | O | O | O |

**What is the expected travel time from your home to the nearest OOH primary care, using your usual means of transport (public or private)?** *(Please only mark one answer)*

Less than 15 minutes

15 to 30 minutes

30 to 60 minutes

More than 60 minutes

Don’t know

**BACKGROUND INFORMATION**

**What is your age?**

Age: ___ years

*Question not in Dutch questionnaire as information was available directly from the consumer panel.*

**What is your sex?**

Male

Female

**Do you live together with another adult?** *(Please give one or more answers)*

No

Yes, with friend(s)s or roommate(s)

Yes, with adult child(ren)

Yes, with wife/husband, partner

Yes, with parent(s)

Yes, in nursing home

Yes, other. *Please describe:* _______________________________________________________________

**How many children do you have (including children for whom you are sharing care)?**

Number of children: ______

1. **What is the age of you oldest and youngest child (in years and months - for children above 3 years, year is sufficient)**

Your oldest child: ....... years and ...... months

Your youngest child: ......... years and ......... months

**In general, how easily can you arrange day care for your child in case of illness?** *(Please only mark one answer)*

Very easily

Easily

With difficult

Very great difficult

Not relevant

Don’t know

**In general, how would you describe your own health?** *(Please only mark one answer)*

Very good

Good

Fair

Bad

Very bad

**In general, how would you describe your child’s health?** *(Please only mark one answer)*

Very good

Good

Fair

Bad

Very bad

**What is the highest educational level that you have completed?** *(Please only mark one answer)*

- No education
- Primary school
- Lower secondary school
- Higher secondary school
- College – bachelor’s degree
- University – bachelor’s degree
- University – master’s degree
- PhD/doctoral
- Other. *Please describe:* ______________________________________________________________

*Answering categories were adjusted to the education system of each country.*

*Question not in Dutch questionnaire as information was available directly from the consumer panel.*

**What is your current job position?** *(Please only mark one answer – in case more answers apply, please mark the most accurate answer)*

- Employed
- Unemployed
- Pre-pension/ pension
- Care for family and household
- Leave
- Disabled
- Student
- Other. *Please describe:* ______________________________________________________________

*Question not in Dutch questionnaire as information was available directly from the consumer panel.*

1. **From which country of birth are you and your parents?** *(Please only mark one cross in each row)*

|  | **Denmark/The Netherlands** | **Other, please write the country** |
| --- | --- | --- |
| You | O | O ______ |
| Your mother | O | O ______ |
| Your father | O | O ______ |

**Do you have a medical education?** *(Please only mark one answer)*

No

Yes, I am a doctor

Yes, I am a nurse

Yes, I have had another medical education. *Please describe:* _____________________________________

1. **Do you use healthcare applications (apps) or the Internet (e.g. ‘Google search’) when you experience a health problem**? *(Please only mark one answer)*

Often

Sometimes

Rarely

Never 🡪 skip question 29

Don’t know 🡪 skip question 29

1. **In general, does using apps or the Internet (e.g. ‘Google search’) influence your need to contact healthcare professionals when you experience a health problem**? *(Please only mark one answer)*

No

Yes, it mostly increases my need to contact

Yes, it sometimes increases and sometimes decrease my need to contact

Yes, it mostly decreases my need to contact

Don’t know

**COMMENTS**

You are welcome to write your comments on the questionnaire here:

_______________________________________________________________________________________

_______________________________________________________________________________________

_______________________________________________________________________________________
