# Supplementary material for: Factors related to out-of-hours help-seeking for acute health problems: a survey study using case scenarios
Source: BMC Public Health. 2019 Jan 8;19:33. doi: 10.1186/s12889-018-6332-6 (PMC6323727; doi:10.1186/s12889-018-6332-6)
Supplement: Supplementary file 2 — Questionnaire for adults. (DOCX 43 kb) [file 12889_2018_6332_MOESM2_ESM.docx]

**Additional file 2. Questionnaire for adults**

*In the English version of the questionnaires, the terms out-of-hours primary care, emergency department, and 112 ambulance care are used in the response categories. The wording was culturally adapted in the translated questionnaires to match the services available for the target group.*

**SITUATION DESCRIPTIONS**

We present six fictive situations. Each of the situations describes an invented case including a health problem affecting your health occurring outside the office hours of your own GP. Please answer what action(s) you would most likely take in this situation at this moment.

We would like to know what you would choose to do in the given situation (i.e. which actions you would most likely take). You do not have to consider what would be the “right” thing to answer or what other people think you should do.

| **Case 1**  *Time:* Sunday at 3 PM.  *Situation:* When you woke up this morning, your left leg was swollen and painful. The leg has a warm, red and painful area with a 10 cm diameter. You do not feel well. You are not sure whether you have a fever. You did not hit your leg. |
| --- |

1. **What would you do, at this moment?** *(Please give one or more answers)*

- Wait and see (no contact with a doctor or similar)
- Self-care (for example a pain killer)
- Ask your partner, a relative, or others for advice
- Check a medical reference book, the internet or an app (for example “Patienthåndbogen”/”Moet ik naar de dokter?”)
- Contact own general practitioner the next working day
- Contact the out-of-hours primary care outside opening hours own GP
- Contact the emergency department
- Call 112 ambulance care
- Do something else *Please describe:* ___________________________________________________

| **Case 2**  *Time:* Monday at 8 PM.  *Situation:* You have been suffering from a severe stomach ache that started suddenly two hours ago; something you have never had before. The pain seems to be localised in your upper stomach, radiating towards your shoulder blades. You have an urge to move around a lot, and you feel nauseous, but you do not vomit. You have had normal defecation patterns all day. |
| --- |

1. **What would you do, at this moment?** *(Please give one or more answers)*

- Wait and see (no contact with a doctor or similar)
- Self-care (for example a pain killer)
- Ask your partner, a relative, or others for advice
- Check a medical reference book, the internet or an app (for example “Patienthåndbogen”/”Moet ik naar de dokter?”)
- Contact own general practitioner the next working day
- Contact the out-of-hours primary care outside opening hours own GP
- Contact the emergency department
- Call 112 ambulance care
- Do something else *Please describe:* ___________________________________________________

| **Case 3**  *Time:* Wednesday at 6 PM.  *Situation:* This morning you suddenly got a severe back pain when lifting a bag with groceries. The pain is continuously present in your lower back. The pain does radiate to your left buttocks, and it limits your movements. You have taken paracetamol (Panadol), but this does not relieve the pain. |
| --- |

1. **What would you do, at this moment?** *(Please give one or more answers)*

- Wait and see (no contact with a doctor or similar)
- Self-care (for example a pain killer)
- Ask your partner, a relative, or others for advice
- Check a medical reference book, the internet or an app (for example “Patienthåndbogen”/”Moet ik naar de dokter?”)
- Contact own general practitioner the next working day
- Contact the out-of-hours primary care outside opening hours own GP
- Contact the emergency department
- Call 112 ambulance care
- Do something else *Please describe:* ___________________________________________________

| **Case 4**  *Time:* Thursday at 7 PM.  *Situation:* You have been suffering from a severe sore throat for two days. You are also coughing slightly and feel feverish. You can take liquids, but swallowing is painful. You have to attend a wedding of a relative in two days. |
| --- |

1. **What would you do, at this moment?** *(Please give one or more answers)*

- Wait and see (no contact with a doctor or similar)
- Self-care (for example a pain killer)
- Ask your partner, a relative, or others for advice
- Check a medical reference book, the internet or an app (for example “Patienthåndbogen”/”Moet ik naar de dokter?”)
- Contact own general practitioner the next working day
- Contact the out-of-hours primary care outside opening hours own GP
- Contact the emergency department
- Call 112 ambulance care
- Do something else *Please describe:* ___________________________________________________

| **Case 5**  *Time:* Wednesday at 7 PM.  *Situation:* You accidently stepped on a piece of glass with your left foot 30 minutes ago. The piece of glass seems to have come out. The bleeding seems to have lessened. The wound is about 3 cm long and is 1-2 mm broad. Your tetanus vaccination is up to date. |
| --- |

1. **What would you do, at this moment?** *(Please give one or more answers)*

- Wait and see (no contact with a doctor or similar)
- Self-care (for example put a plaster on)
- Ask your partner, a relative, or others for advice
- Check a medical reference book, the internet or an app (for example “Patienthåndbogen”/”Moet ik naar de dokter?”)
- Contact own general practitioner the next working day
- Contact the out-of-hours primary care outside opening hours own GP
- Contact the emergency department
- Call 112 ambulance care
- Do something else *Please describe:* ___________________________________________________

| **Case 6**  *Time:* Saturday at 4 PM.  *Situation:* Your left foot was twisted yesterday when you were walking in the forest. Your left ankle was directly painful and swollen. Initially, you were able to walk on the injured foot, but now you are unable to even rest on it. Your left ankle is quite painful and seems swollen compared to the right one. |
| --- |

1. **What would you do, at this moment?** *(Please give one or more answers)*

- Wait and see (no contact with a doctor or similar)
- Self-care (for example put ice on)
- Ask your partner, a relative, or others for advice
- Check a medical reference book, the internet or an app (for example “Patienthåndbogen”/”Moet ik naar de dokter?”)
- Contact own general practitioner the next working day
- Contact the out-of-hours primary care outside opening hours own GP
- Contact the emergency department
- Call 112 ambulance care
- Do something else *Please describe:* ___________________________________________________

**FACTORS AFFECTING DECISION-MAKING**

The next questions relate to general factors that may affect decision-making regarding health problems.

1. **We are interested in how you feel about the following statements. Read each statement carefully. Indicate how you feel about each statement.** *(Please mark one answer per statement)*

|  |  | **Not at all true** | **Hardly true** | **Moderately true** | **Exactly true** |
| --- | --- | --- | --- | --- | --- |
| 1. | I can always manage to solve difficult problems if I try hard enough | O | O | O | O |
| 2. | If someone opposes me, I can find the means and ways to get what I want | O | O | O | O |
| 3. | It is easy for me to stick to my aims and accomplish my goals | O | O | O | O |
| 4. | I am confident that I could deal efficiently with unexpected events | O | O | O | O |
| 5. | Thanks to my resourcefulness, I know how to handle unforeseen situations | O | O | O | O |
| 6. | I can solve most problems if I invest the necessary effort | O | O | O | O |
| 7. | I can remain calm when facing difficulties because I can rely on my coping abilities | O | O | O | O |
| 8. | When I am confronted with a problem, I can usually find several solutions | O | O | O | O |
| 9. | If I am in trouble, I can usually think of a solution | O | O | O | O |
| 10. | I can usually handle whatever comes my way | O | O | O | O |

*We used validated Danish, Dutch, and German versions of the Generalized Self-Efficacy scale [1, 2].*

1. **Over the last two weeks, how often have you been bothered by the following problems?**

|  |  | **Not at all** | **Several days** | **More than half the days** | **Nearly every day** |
| --- | --- | --- | --- | --- | --- |
| 1. | Feeling nervous, anxious or on edge | O | O | O | O |
| 2. | Not being able to stop or control worrying | O | O | O | O |

*We used validated Danish, Dutch, and German versions of the Generalized Anxiety Disorder scale (GAD-2) [3, 4].*

**Do you have somebody to talk to if you have problems or you need support?** *(Please only mark one answer)*

No, never or almost never

Yes, sometimes

Yes, often

Yes, mostly

We used two scales of the validated Health Literacy Questionnaire (HLQ). As the HLQ is copyrighted to Deakin University, publication of the items or scales is not permitted [5]*.*

1. **How severe would your medical problem have to be before you felt it was appropriate to contact …?** *(Please mark one grade per row)*

|  | **Not severe** | | | |  |  |  |  | **Very severe** | | | **Don’t know** |
| --- | --- | --- | --- | --- | --- | --- | --- | --- | --- | --- | --- | --- |
| … your own GP | **0** | **1** | **2** | **3** | **4** | **5** | **6** | **7** | **8** | **9** | **10** | O |
| … OOH primary care | **0** | **1** | **2** | **3** | **4** | **5** | **6** | **7** | **8** | **9** | **10** | O |
| … 112 | **0** | **1** | **2** | **3** | **4** | **5** | **6** | **7** | **8** | **9** | **10** | O |

1. **The following statements concern your considerations for contacting OOH-PC. Please answer to which degree you agree with each statement.** *(Please mark one answer per statement)*

|  |  | **Totally agree** | **Agree** | **Not agree and not disagree** | **Disagree** | **Totally disagree** | **Don’t know** |
| --- | --- | --- | --- | --- | --- | --- | --- |
| 1. | The OOH primary care is intended for all medical problems (including non-urgent problems) that occur outside my GP’s normal opening hours | O | O | O | O | O | O |
| 2. | I can contact OOH primary care at any time, because it is financed by taxation (Denmark)/my insurance (the Netherlands, Switzerland) | O | O | O | O | O | O |
| 3. | I feel more personal barriers in relation to contacting OOH primary care than contacting my own GP during daytime | O | O | O | O | O | O |
| 4. | I carefully consider whether I should contact OOH primary care, because I do not want to disturb the health professionals | O | O | O | O | O | O |

**In the past year, how many times have you contacted the following health care providers regarding yourself and/or your children?** *(Please only mark one cross in each row– if you are unsure, please answer what you think is most accurate)*

|  | **Never** | **1** | **2** | **3** | **4** | **5 or more** | **Don’t know/ not relevant** |
| --- | --- | --- | --- | --- | --- | --- | --- |
| Own GP | O | O | O | O | O | O | O |
| OOH primary care | O | O | O | O | O | O | O |
| Emergency department | O | O | O | O | O | O | O |
| 112 | O | O | O | O | O | O | O |

**How satisfied are you in general with the following health care providers?** *(Please only mark one cross in each row)*

|  | **Very satisfied** | **Satisfied** | **Not satisfied, not satisfied** | **Dissatisfied** | **Very dissatisfied** | **Don’t know** | **Not relevant/ no contact** |
| --- | --- | --- | --- | --- | --- | --- | --- |
| Own GP | O | O | O | O | O | O | O |
| OOH primary care | O | O | O | O | O | O | O |
| Emergency department | O | O | O | O | O | O | O |
| 112 | O | O | O | O | O | O | O |

**During the last two years, have you experienced practical problems in contacting your own GP during day time, due to …** *(Please only mark one cross in each row)*

|  | **No problems** | **Yes, few problems** | **Yes, some problems** | **Yes, many problems** | **Don’t know** | **Not relevant** |
| --- | --- | --- | --- | --- | --- | --- |
| … your own working hours or private appointments? | O | O | O | O | O | O |
| … your GPs telephone accessibility? | O | O | O | O | O | O |
| ...the possibility to make a telephone appointment with your GP? | O | O | O | O | O | O |
| … your GPs availability for a clinic appointment? | O | O | O | O | O | O |
| ...the accessibility to your own GP practice by website (i.e. making an appointment, repeat prescription, asking questions)? | O | O | O | O | O | O |

**What is the expected travel time from your home to the nearest OOH primary care, using your usual means of transport (public or private)?** *(Please only mark one answer)*

Less than 15 minutes

15 to 30 minutes

30 to 60 minutes

More than 60 minutes

Don’t know

**BACKGROUND INFORMATION**

**What is your age?**

Age: ___ years

*Question not in Dutch questionnaire as information was available directly from the consumer panel.*

**What is your sex?**

Male

Female

*Question not in Dutch questionnaire as information was available directly from the consumer panel.*

**Do you live together with another adult?** *(Please give one or more answers)*

No

Yes, with friend(s)s or roommate(s)

Yes, with adult child(ren)

Yes, with wife/husband, partner

Yes, with parent(s)

Yes, in nursing home

Yes, other. *Please describe:* _____________________________________________________

**In general, how would you describe your own health?** *(Please only mark one answer)*

Very good

Good

Fair

Bad

Very bad

**What is the highest educational level that you have completed?** *(Please only mark one answer)*

- No education
- Primary school
- Lower secondary school
- Higher secondary school
- College – bachelor’s degree
- University – bachelor’s degree
- University – master’s degree
- PhD/doctoral
- Other. *Please describe:* ______________________________________________________________

*Answering categories were adjusted to the education system of each country.*

*Question not in Dutch questionnaire as information was available directly from the consumer panel.*

**What is your current job position?** *( Please only mark one answer - in case more answers apply, please mark the most accurate answer)*

- Employed
- Unemployed
- Pre-pension/ pension
- Care for family and household
- Leave
- Disabled
- Student
- Other. *Please describe:* ______________________________________________________________

*Question not in Dutch questionnaire as information was available directly from the consumer panel.*

1. **From which country of birth are you and your parents?** *(Please only mark one cross in each row)*

|  | **Denmark/The Netherlands/Switzerland** | **Other, please write the country** |
| --- | --- | --- |
| You | O | O ______ |
| Your mother | O | O ______ |
| Your father | O | O ______ |

**Do you have a medical education?** *(Please only mark one answer)*

No

Yes, I am a doctor

Yes, I am a nurse

Yes, I have had another medical education. *Please describe:* ____________________________________

1. **Do you use healthcare applications (apps) or the Internet (e.g. ‘Google search’) when you experience a health problem**? *(Please only mark one answer)*

Often

Sometimes

Rarely

Never 🡪 skip question 25

Don’t know 🡪 skip question 25

1. **In general, does using apps or the Internet (e.g. ‘Google search’) influence your need to contact healthcare professionals when you experience a health problem**? *(Please only mark one answer)*

No

Yes, it mostly increases my need to contact

Yes, it sometimes increases and sometimes decrease my need to contact

Yes, it mostly decreases my need to contact

Don’t know

Not relevant – rarely/never use this

*The Swiss questionnaire had four extra questions concerning ethnicity, being listed at a GP, and the insurance model.*

**COMMENTS**

You are welcome to write your comments on the questionnaire here:

_______________________________________________________________________________________

_______________________________________________________________________________________

_______________________________________________________________________________________
