# Supplementary material for: Factors related to out-of-hours help-seeking for acute health problems: a survey study using case scenarios
Source: BMC Public Health. 2019 Jan 8;19:33. doi: 10.1186/s12889-018-6332-6 (PMC6323727; doi:10.1186/s12889-018-6332-6)
Supplement: Supplementary file 3 — Categorisation of influential factors. (DOCX 24 kb) [file 12889_2018_6332_MOESM3_ESM.docx]

**Additional file 3. Categorisation of influental factors**

| **Factors** | **Final categories** | **Categorisation** |
| --- | --- | --- |
| **PREDISPOSING** | | |
| **Age** | Continuous |  |
| **Gender** | Male *(ref)* | ‘Male’ |
|  | Female | ‘Female’ |
| **Education level** | Low | ≤ 10 years |
|  | Middle | >10 & ≤ 15 years |
|  | High *(ref)* | > 15 years |
|  |  | *This categorisation was made following the ISCED guidelines[6]* |
| **Medical education** | None *(ref)* | ’None’ |
|  | Some/nurse/doc | ’Doctor’, ’Nurse’, ’Other’ |
| **Ethnicity** | Native *(ref)* | Both parents born in the study country |
|  | Western migrant | At least one parent born in a European country (except Turkey), North America, Oceania, Indonesia, or Japan* |
|  | Non-western migrant | At least one parent born in another country* |
|  |  | **If the parents were born in different country groups outside the study countries, mother’s country of birth was used to determine ethnicity* |
| **Employment** | Unemployed | ‘Disabled’, ‘Unemployed, seeking work, social security’, ‘Pre-pension (not because of health problems)’, ‘Pension’, ‘Student’, ‘Care for family and household (without social security)’ |
|  | Employed *(ref)* | ‘Employed’, ‘Self-employed’ |
| **Living status** | Living alone | ‘No’ |
|  | Living with another adult *(ref)* | ‘Yes, with friend(s) or roommate(s)’, ‘Yes, with adult child(ren)’, ‘Yes, with wife/husband, partner’, ‘Yes, with parent(s)’, ‘Yes, in nursing home’, ‘Yes, other’ |
| **Number of children^1^** | One | 1 |
|  | More than one *(ref)* | ≥ 1 |
| **Social support** | Lacking social support | ‘No, never or almost never’, ‘Yes, sometimes’ |
|  | Social support *(ref)* | ‘Yes, often’, ‘Yes, mostly’ |
| **Health literacy – navigating the system** | Low ability  Middle ability  High ability  Highest ability *(ref)* | Mean of 6 items < 2.5 |
|  |  | Mean of 6 items ≥ 2.5 & < 3.5 |
|  |  | Mean of 6 items ≥ 3.5 & < 4.5 |
|  |  | Mean of 6 items ≥ 4.5 & ≤ 5.0 |
|  |  | *6 items, with 5-point scale. At least three valid answers were needed* |
| **Health literacy – finding information** | Low ability  High ability  Highest ability *(ref)* | Mean of 4 items < 2.5 |
|  |  | Mean of 4 items of scale ≥ 2.5 & < 3.5 |
|  |  | Mean of 4 items of scale ≥ 3.5 & ≤ 4 |
|  |  | *4 items, with 5-point scale. At least two valid answers were needed* |
| **Self-efficacy** | Low | Sum score < mean sum score GSE-10 |
|  | High *(ref)* | Sum score > mean sum score GSE-10 |
|  |  | *10 items, with 4-point scale. At least 7 valid answers were needed* |
| **Anxiety** | No anxiety *(ref)* | Sum score 2 items ≥ 3 |
|  | Anxiety | Sum score 2 items < 3 |
|  |  | *Two valid answers* |
| **Attitude towards use of out-of-hours primary care** | Low barrier  High barrier *(ref)* | No missing value and sum score ≥ 6, one missing value and sum score ≥ 5  No missing value and sum score ≤ 5, one missing value and sum score ≤ 4  *4 statements, with 5-point scale; per item score reflected range from feeling barrier to having right. Scores per item were dichotomised and summed; score range: 4-8. At least 3 valid answers were needed* |
| **ENABLING** | | |
| **Travel time** | <15 minutes *(ref)* | ‘Less than 15 minutes’ |
|  | 15-30 minutes | ‘15 to 30 minutes’ |
|  | >30 minutes | ‘30 to 60 minutes’, ‘More than 60 minutes’ |
| **Problems – own work or private appointments** | No/few problems *(ref)* | ‘No, no problems’, ‘Yes, few problems’ |
|  | Some/many problems | ‘Yes, some problems’, ‘Yes, many problems’ |
| **Problems – organising childcare^1^** | Easy *(ref)* | ‘Very easily’, ‘Easily’ |
|  | Difficult | ‘With difficult’, ‘Very great difficult’ |
| **Problems – accessibility of own GP** | No/few problems *(ref)* | ‘No, no problems’, ‘Yes, few problems’ |
|  | Some/many problems | ‘Yes, some problems’, ‘Yes, many problems’ |
| **Problems – availability of own GP** | No/few problems *(ref)* | ‘No, no problems’, ‘Yes, few problems’ |
|  | Some/many problems | ‘Yes, some problems’, ‘Yes, many problems’ |
| **NEED** | | |
| **Self-assessed health child/adult** | Poor | ‘Very bad’, ‘Bad’ |
|  | Good *(ref)* | ‘Fair’, ‘Good’, ‘Very good’ |
| **BEHAVIOUR** | | |
| **Frequency of contacts to own GP** | None/one contact *(ref)* | 0-1 contacts |
|  | Few contacts | 2-4 contacts |
|  | More contacts | ≥ 5 contacts |
| **Frequency of contacts to out-of-hours care** | None *(ref)* | Sum score on 3 variables (contact frequency out-of-hours primary care, ED and 112) = 0 |
|  | One contact | Sum score on 3 variables (contact frequency out-of-hours primary care, ED and 112) = 1 |
|  | More contacts | Sum score on 3 variables (contact frequency out-of-hours primary care, ED and 112) > 1 |
|  |  | *At least three valid answers* |

*^1^ Only included in questionnaire for parents; ref = reference category in regression analyses.*

**References**

1. **General Self-Efficacy Scale (GSE)** [<http://userpage.fu-berlin.de/health/selfscal.htm> ]

2. Schwarzer R, Jerusalem M: **Generalized Self-Efficacy scale**. In: *J Winman, S Wright, M Johnston Measures in health psychology: A user's portfolio Causal and control beliefs.* edn. Windsor, England: NFER-NELSON; 1995: 35-37.

3. **Screener Overview** [<https://www.phqscreeners.com/select-screener>]

4. Kroenke K, Spitzer RL, Williams JB, Monahan PO, Lowe B: **Anxiety disorders in primary care: prevalence, impairment, comorbidity, and detection**. *Ann Intern Med* 2007, **146**(5):317-325.

5. Osborne RH, Batterham RW, Elsworth GR, Hawkins M, Buchbinder R: **The grounded psychometric development and initial validation of the Health Literacy Questionnaire (HLQ)**. *BMC Public Health* 2013, **13**:658.

6. UNESCO Institute for Statistics: **International standard classification of education: ISCED 2011**. In*.* Montreal: UNESCO Institute for Statistics; 2012.
